# Analysis of the dynamic correlation between chronic comorbidities and health shocks among middle-aged and older adults people in rural mountainous areas of southern Ningxia—from 14 years of follow-up panel data

**DOI:** 10.3389/fpubh.2025.1599656

**Published:** 2025-06-23

**Authors:** Juan Yang, Shan Liu, Fei Li, Haodong Meng, Hui Qiao, Yongxin Xie

**Affiliations:** ^1^School of Public Health, Ningxia Medical University, Yinchuan, China; ^2^Key Laboratory of Environmental Factors and Chronic Disease Control, Yinchuan, China

**Keywords:** chronic comorbidities, health shocks, dynamic correlation, middle-aged and older adults people, developmental trajectory

## Abstract

**Background:**

With the acceleration of the aging process, the health shock on the middle-aged and older adults people has become a key issue that urgently needs to be solved. Health shock refers to the long-term fluctuating effect on the future level of family health welfare due to the uncertainty of the health status of family members in the short term. The occurrence of health shocks is related to many factors, among which chronic comorbidity is an important factor affecting the occurrence of health shocks in the middle-aged and older adults people. The purpose of this study was to explore the developmental trajectories and interactions of chronic comorbidities and health shocks in rural residents in the southern mountainous areas of Ningxia.

**Methods:**

On the basis of health follow-up data from rural middle-aged and older adults people in Ningxia in 2009, 2015, 2019 and 2022, the dynamic associations between chronic comorbidities and health shocks in rural residents in mountainous areas of southern Ningxia were analyzed via the latent growth model (LGM) and cross-lagged model (CLM).

**Results:**

The unconditional latent growth model (ULGM) revealed that chronic comorbidities (*χ*^2^ = 26.807, *p* < 0.001) and health shocks (*χ*^2^ = 64.296, *p* < 0.001) are increasing in the 14-year period from 2009 to 2022. The parallel latent growth model (PLGM) revealed that the initial level of health shock had a significant positive predictive effect on both the initial level and the rate of change of chronic comorbidities. The change rate of health shocks had a significant positive predictive effect on the initial level of chronic comorbidities and a relatively significant negative predictive effect on the rate of change of chronic comorbidities. The initial level of chronic comorbidities had a significant positive predictive effect on the initial level of health shock and a significant negative predictive effect on the change rate of health shock. The change rate of chronic comorbidity has no significant predictive effect on the initial level and change rate of health shock. The results of the cross-lagged model (CLM) indicate a bidirectional causal association between chronic comorbidities and health shocks.

**Conclusion:**

Based on a 14-year tracking data study, this research found that both chronic comorbidities and health shocks were on the rise, and there was a dynamic interaction and bidirectional causal relationship between the two, which could predict each other’s development trends. Based on the empirical results, it is recommended to strengthen the monitoring and management chronic comorbidities to reduce the risk of health shocks. Meanwhile, it is also necessary to closely monitor health shock events, and accordingly optimize the management strategies for chronic comorbidities, thereby reducing the incidence of health shocks among the middle-aged and older adults population, improving the quality of life and health, and promoting the realization of the goal of healthy aging.

## 1 Introduction

Today, the world is facing the severe challenge of population aging (1), especially in China (2). As one of the countries with the largest population in the world, China is becoming a rapidly aging country with an extension of average life expectancy (3). According to the data of the “seventh census,” the older adults population over the ages of 60 and 65 years accounted for 18.7 and 13.5%, respectively, of the total population in China. Moreover, with China’s middle-aged people in their 40s and 50 s gradually entering the aging army, the China Development Foundation published a research report on aging in 2020, which estimated that China will account for 14% of the total population over the age of 65 before and after 2022, ranking among the ranks of the aging society (4, 5). With the acceleration of China’s aging, a series of challenges have emerged, among which health shocks to the middle-aged and older adults populations have become key issues that need to be solved urgently.

At present, there is no clear definition of health shocks at home and abroad. Chinese scholars usually take factors such as “self-rated health status,” “being informed of illness by doctors,” “suffering from new acute or chronic diseases or death,” “significant medical and health outcomes,” and “weight loss” as indicators to measure health shocks (6–8). Most scholars abroad have focused their research on health shocks on major disease indicators such as “the number of days when normal activities and work cannot be carried out due to illness,” “the main diseases that cause the first hospital admission within a year,” and “newly diagnosed cancers, heart diseases, and strokes” (9, 10). This study integrates relevant domestic and foreign literature and combines existing data and research. On the basis of fully considering the factors of time range and content range, and referring to the definition of health shock by Ai Xiaoqian et al., the following definition is made: (1) Within 2 weeks before the investigation, taking a break from work, study or being bedridden for 1 day or more due to discomfort Moreover, the total self-paid medical expenses for this medical treatment (including transportation, accommodation, meals, accompanying care and other expenses) account for more than 10% of the family’s annual income. (2) The proportion of healthcare expenditure spent throughout the year to the family’s annual income is greater than 40%. Health shocks cause considerable harm to middle-aged and older adults people (11–13), which can lead to a decline in physiological function, increase the economic burden on families or affect the mental health of middle-aged and older adults people; thus, it is particularly necessary to understand the path of health shocks. A review of the relevant literature revealed that chronic disease is an important factor affecting the health of middle-aged and older adults people (14). The prevalence of chronic diseases in the United States has remained high in recent decades, with half of American adults living with at least one chronic disease in 2012 and at least one in four living with two or more chronic conditions (15), known as chronic comorbidities. Chronic comorbidities refer to two or more chronic noncommunicable diseases (3, 16), which are far more harmful to middle-aged and older adults people than a single chronic disease. Relevant domestic studies have shown that the prevalence of chronic diseases in China’s aging population is increasing rapidly (3), and the chronic disease pattern has changed from a single pattern to a multidisease coexistence pattern.

This study aims to explore the dynamic correlation between chronic comorbidities and health shocks, the developmental trajectory of chronic comorbidities and health shocks, and the causal relationship between the two using 14-year follow-up data from Ningxia from 2009 to 2022. The results of this study can provide scientific guidance and data support for the prevention and treatment of chronic comorbidities and the reduction of health shocks.

## 2 Data and methods

### 2.1 Data source

This study selected 14 years and 4 periods of health follow-up data for analysis from 2009 to 2022. The method of multistage stratified cluster random sampling was adopted to obtain the research objects. After informed consent was obtained from all the subjects, a health-related questionnaire was administered. This study was approved by the Ethics Committee of Ningxia Medical University (approval number: 2022-G165).

### 2.2 Research objects and methods

**Figure 1:**
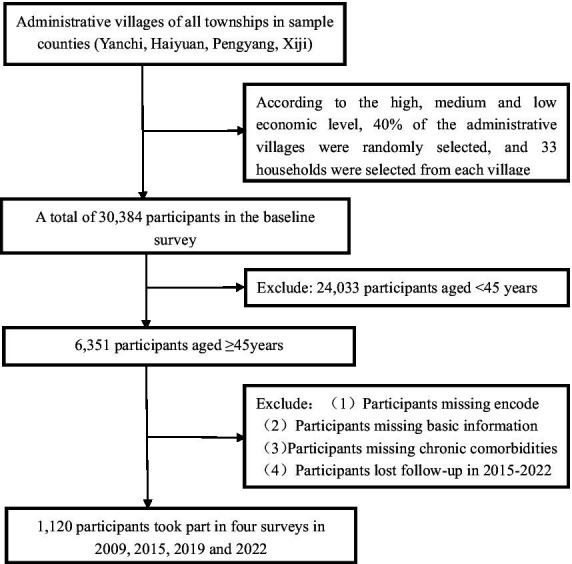
Flow chart of participant selection.

Figure 1 shows the selection process for study participants. In this study, 14 years and 4 periods of health follow-up data were used to obtain data from the study subjects via multistage stratified cluster random sampling. All the administrative villages in the towns of the sample counties (Yanchi, Haiyuan, Pengyang, Xiji) were divided into three levels according to high, medium and low economic levels. 40% of the administrative villages were randomly selected from each level. 33 rural household households were selected from each village by the systematic sampling method. A “face-to-face” questionnaire survey was conducted among all middle-aged and older adults people aged 45 and above in the household households. The survey contents include general demographic characteristics, indicators related to family economic conditions, health-related indicators, indicators related to the utilization of health services, and indicators related to health poverty alleviation policies. With 2009 as the baseline survey and the deletion of missing values for important variables, a total of 6,351 subjects were included in the study, and follow-up was conducted in 2015, 2019 and 2022, with personal codes used as the matching variables. Finally, 1,120 subjects participated in the four phases of the survey.

### 2.3 Index measurements

#### 2.3.1 Chronic comorbidity index

By consulting relevant domestic and foreign literatures (17–19), the Charlson Comorbidity Index (CCI) is currently the most widely used comorbidity assessment tool. Charlson et al. developed CC1 in 1987 by referring to the relative risk of different diseases for the one-year mortality rate of patients, and subsequently incorporated the assessment of age factors. The CCI consists of three major parts: disease assessment, severity assessment, and scoring system. Among them, disease assessment includes 19 diseases, and severity assessment assigns weights of 1, 2, 3, and 6 points, respectively, based on their severity. The CCI adjusts the scoring based on age. Starting from the age of 50 to 59, 1 point is awarded. For every additional 10 years of age, the score increases by 1 point. In this study, comorbidity scoring criteria were defined based on the CCI subscale, the age-adjusted CCI subscale, and combined with the multi-year follow-up data of the research group. The specific assignment values are shown in Table 1. The Charlson comorbidity index is ultimately composed of the scores of different diseases plus the corresponding scores of different ages, which is used to quantify the severity of chronic comorbidity. The higher the score, the more severe the degree of chronic comorbidity.

**Table 1:** aCCI score sheet.

(A) Grading table for different types of diseases	
Disease	Score
Myocardial infarction	1
Congestive heart failure	1
Peripheral vascular disease	1
Dementia	1
Cerebrovascular disease	1
Connective tissue disease	1
Peptic ulcer	1
Diabetes	1
Chronic obstructive pulmonary disease	1
Mild liver disease	1
Hypertension	1
Depression	1
Moderate to severe chronic kidney disease	2
Diabetes mellitus with organ damage	2
Solid tumor	2
Leukemia	2
Malignant lymphoma	2
Skin ulcers/cellulitis	2
Liver disease	3
Malignant tumor	6

(B) Different age grading tables	
Age (years)	Score
<50	0
50–59	1
60–69	2
70–79	3
80–89	4
90–99	5

#### 2.3.2 Health shock intensity

Ai Xiaoqian et al. (14) were used as a reference to define the intensity of health shocks. The intensity of the health shock was measured via the distance between total out-of-pocket medical expenditures and 10 percent of annual household income and the distance between annual household health expenditures and 40 percent of annual household income. To avoid the influence of individual extreme values on the results, the range of health shock intensity was defined as 0~1. D represents the number of days of absence from work, school or bed due to discomfort in the 2 weeks before the survey; OOP represents the total out-of-pocket medical expenses incurred during the 2 weeks prior to the survey due to poor medical behavior; T1 represents annual household income; and T represents annual household health care spending.

$$\text{Intensity}=\{\begin{array}{c} 0,\text{if}\;D=0\;\text{and}\;\mathrm{OOP}÷T1<0.1\\ \mathrm{OOP}÷T1-0.1,\text{if}\;D\geq 1\;\text{and}\;\mathrm{OPP}÷T1\geq 0.1 \end{array}$$

$$\text{Intensity}=\{\begin{array}{c} 0,\text{if}\;T÷T1<0.4\\ T÷T1-0.4,\text{if}\;T÷T1\geq 0.4 \end{array}$$

### 2.4 Demographic variables

The demographic variables in this study included age, sex (male; female), education level (never attended school; elementary school; middle school; high school or more), marital status (unmarried; married; divorced; widow/widower; other), and family size (1–3 people; 4–6 people; ≥7 people).

### 2.5 Statistical methods

SPSS 27.0 and R4.4.0 were used to clean the data, Mplus 8.3 was used to create the LGM and CLM, and R4.4.0 was used to draw correlation heatmaps. First, the mean ± standard deviation (M ± SD) and frequency (percent) were used to describe the basic information of the study participants. Second, the correlation heatmap between chronic comorbidities and health shocks was drawn using the ggplot2 package, haven package, reshape2 package, Hmisc package and RColorBrewer package in the R4.4.0 statistical software. Then, the LGM of chronic comorbidities and health shocks was created (ULGM; PLGM) to study the developmental trajectory of chronic comorbidities and health shocks. Finally, a CLM was used to study whether there was a causal relationship between chronic comorbidities and health shocks. The test level was 0.05.

## 3 Results

### 3.1 Basic characteristics of the research subjects

A total of 1,120 people participated in the survey in all four phases over 14 years, and the basic characteristics of the research objects were described with the information of the latest phase. The total population was 1,120, of which 192 were 45–59 years old and 928 were ≥60 years old. There were 591 males (52.77%) and 529 females (47.23%). The highest proportion of those who had never attended school was 49.29%. The marital status is the highest at 85.09%. The number of families with 1 to 3 people is the largest, with 701 people, accounting for 62.59% (see Table 2).

**Table 2:** Basic characteristics of middle-aged and older adults people in rural areas of southern Ningxia.

Variables		Total	45–59	≥60
*N*		1,120	192	928
Age (M ± SD)		66.86 ± 7.846	57.74 ± 2.224	68.74 ± 7.248
Gender	Male	591 (52.77%)	106 (55.21%)	485 (52.26%)
	Female	529 (47.23%)	86 (44.79%)	443 (47.74%)
Education	Never attended school	552 (49.29%)	64 (33.33%)	488 (52.59%)
	Elementary school	370 (33.04%)	64 (33.33%)	306 (32.97%)
	Middle school	152 (13.57%)	54 (28.13%)	98 (10.56%)
	High school or more	46 (4.11%)	10 (5.21%)	36 (3.88%)
Marital status	Unmarried	16 (1.43%)	3 (1.56%)	13 (1.40%)
	Married	953 (85.09%)	182 (94.79%)	771 (83.08%)
	Divorced	2 (0.18%)	1 (0.52%)	1 (0.11%)
	Widow/widower	148 (13.21%)	5 (2.60%)	143 (15.41%)
	Other	1 (0.09%)	1 (0.52%)	0 (0.00%)
Household size	1–3	701 (62.59%)	105 (54.69%)	596 (64.22%)
	4–6	319 (28.48%)	70 (36.46%)	249 (26.83%)
	≥7	100 (8.93%)	17 (8.85%)	83 (8.94%)
aCCIT1		1.104 ± 1.032	0.706 ± 0.703	2.476 ± 0.775
aCCIT2		2.042 ± 1.301	1.391 ± 0.917	2.876 ± 1.243
aCCIT3		2.508 ± 1.284	1.432 ± 0.744	3.130 ± 1.108
aCCIT4		2.910 ± 1.238	1.385 ± 0.620	3.225 ± 1.090
HSIT1		0.066 ± 0.167	0.060 ± 0.162	0.089 ± 0.185
HSIT2		0.046 ± 0.155	0.032 ± 0.130	0.064 ± 0.181
HSIT3		0.082 ± 0.190	0.055 ± 0.145	0.098 ± 0.210
HSIT4		0.068 ± 0.174	0.060 ± 0.143	0.070 ± 0.179

### 3.2 Correlation analysis between chronic comorbidities and health shocks

**Figure 2:**
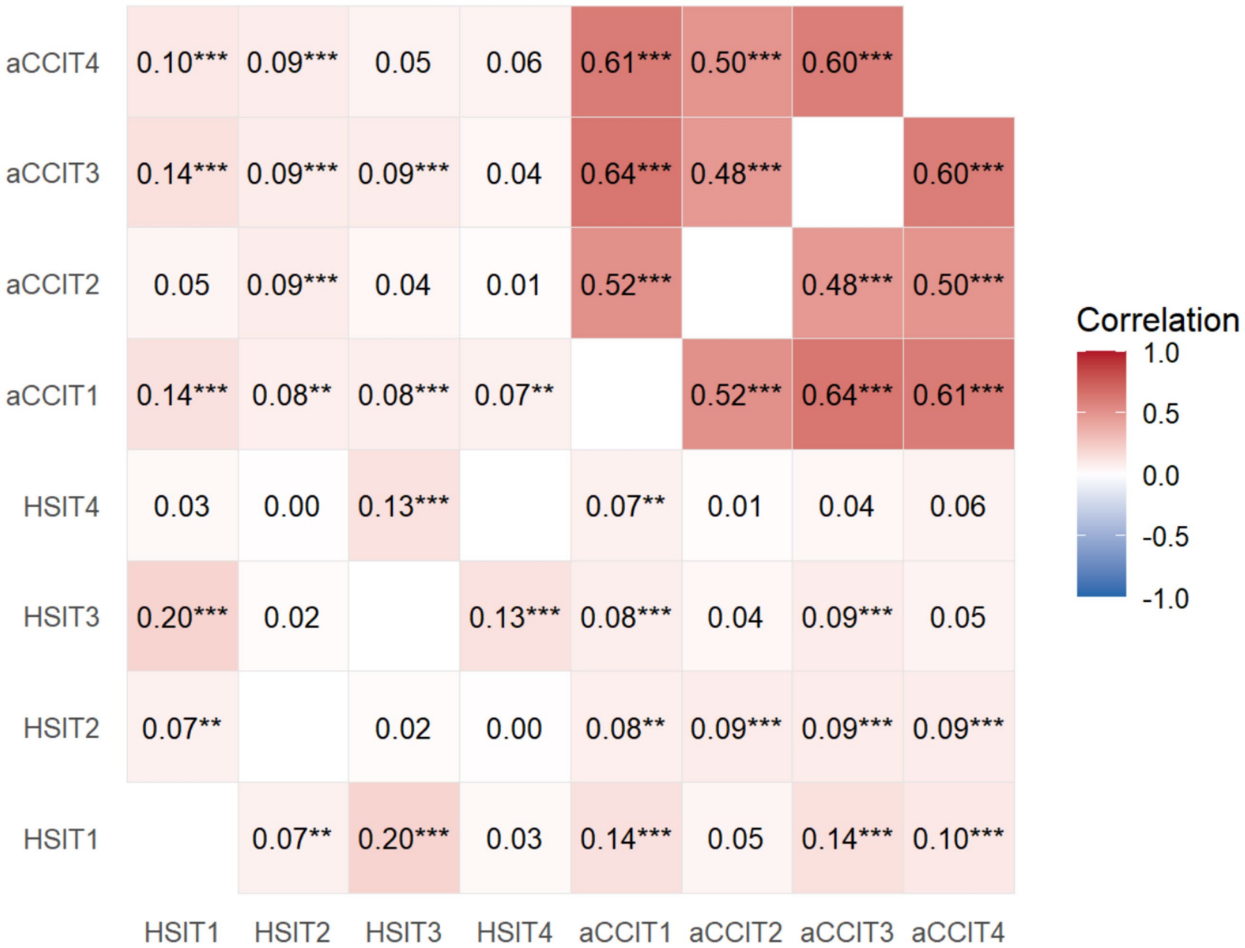
Spearman correlation between chronic comorbidity and health shock at T1, T2, T3, and T4 in the sample population. aCCI, age-adjusted Charlson comorbidity index; HSI, health shock intensity; aCCIT1, age-adjusted Charlson comorbidity index in 2009; aCCIT2, age-adjusted Charlson comorbidity index in 2015; aCCIT3, age-adjusted Charlson comorbidity index in 2019; aCCIT4, age-adjusted Charlson comorbidity index in 2022; HSIT1, health shock intensity in 2009; HSIT2, health shock intensity in 2015; HSIT3, health shock intensity in 2019; HSIT4, health shock intensity in 2022; ^***^p < 0.01, ^**^p < 0.05.

Spearman correlation analysis was performed on the observed data of chronic comorbidities and health shocks at four time points, and the results revealed a significant positive correlation between chronic comorbidities and health shocks at each time point (*p* < 0.05), as shown in Figure 2.

### 3.3 Developmental trajectory of chronic comorbidities and health shocks

**Figure 3:**
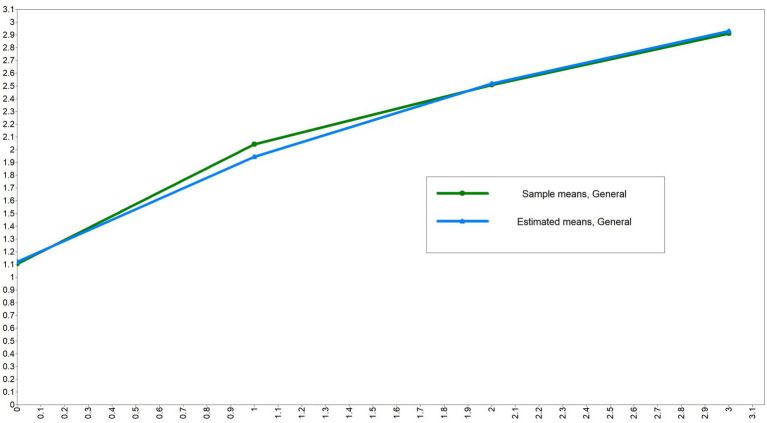
Trends of chronic comorbidities from 2009 to 2022.

**Figure 4:**
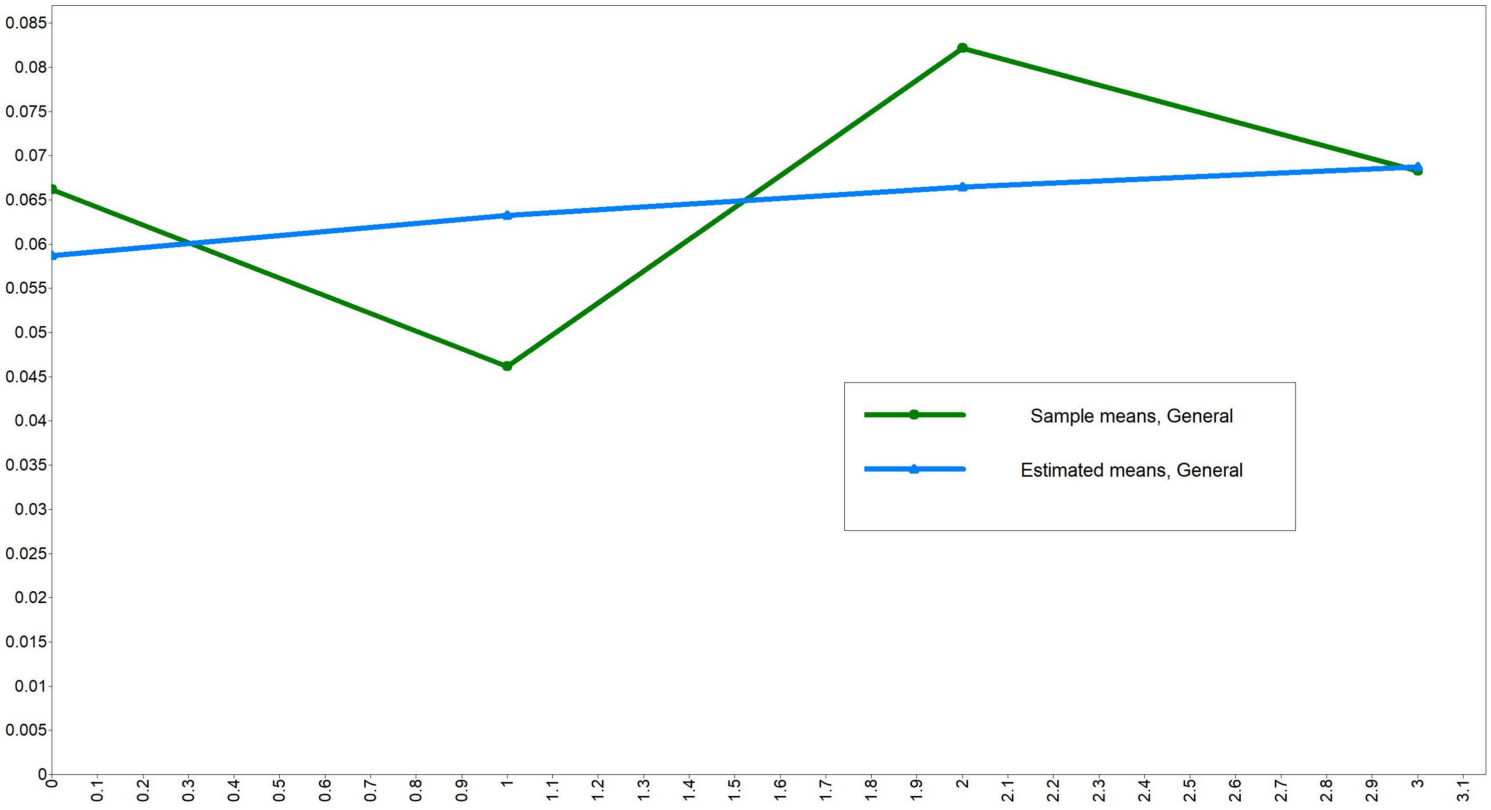
Trends of health shocks from 2009 to 2022.

ULGM was performed for chronic comorbidity and health shock measurements at different time points to examine the developmental trajectories of both. First, a ULGM of chronic comorbidities was established. The model fit results were good (CFI and TLI were both >0.9, SRMR and RMSEA were both <0.08). The initial level of the intercept was shown to be 1.327, and chronic comorbidities increased significantly across the four measurements, as shown in Table 3 and Figure 3. Then, the ULGM of health shocks at the four time points is constructed, and the model fit results are not very satisfactory. The initial level of the health shock intercept was 1.013, and there was a significant negative correlation between the intercept and slope (*β* = −0.636, *p* < 0.05), as shown in Table 3 and Figure 4.

**Table 3:** Unconditional latent growth model of chronic comorbidities and health shocks.

Variable	Model fit indices						Intercept	Slope	Slope with intercept
	*χ* ^2^	*df*	CFI	TLI	RMSEA	SRMR	*β*	*β*	*β*
Chronic comorbidity	26.807	5	0.987	0.985	0.062	0.048	1.327^***^ (0.068)	6.779 (3.722)	0.374 (0.458)
Health shock	64.296	5	0.091	0.000	0.103	0.061	1.013^***^ (0.240)	0.212 (0.207)	−0.636^**^ (0.247)

*** and ** indicate statistical significance at the 1% and 5% levels, respectively, with standard errors reported in parentheses.

### 3.4 Parallel latent growth model for chronic comorbidities and health shocks

**Figure 5:**
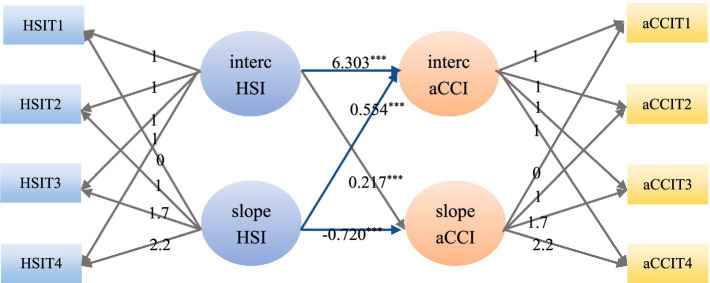
A parallel latent growth model of health shock at the chronic comorbidity level. aCCI, age-adjusted Charlson comorbidity index; HSI, health shock intensity; aCCIT1, age-adjusted Charlson comorbidity index in 2009; aCCIT2, age-adjusted Charlson comorbidity index in 2015; aCCIT3, age-adjusted Charlson comorbidity index in 2019; aCCIT4, age-adjusted Charlson comorbidity index in 2022; HSIT1, health shock intensity in 2009; HSIT2, health shock intensity in 2015; HSIT3, health shock intensity in 2019; HSIT4, health shock intensity in 2022; ^***^p < 0.01, ^**^p < 0.05.

**Figure 6:**
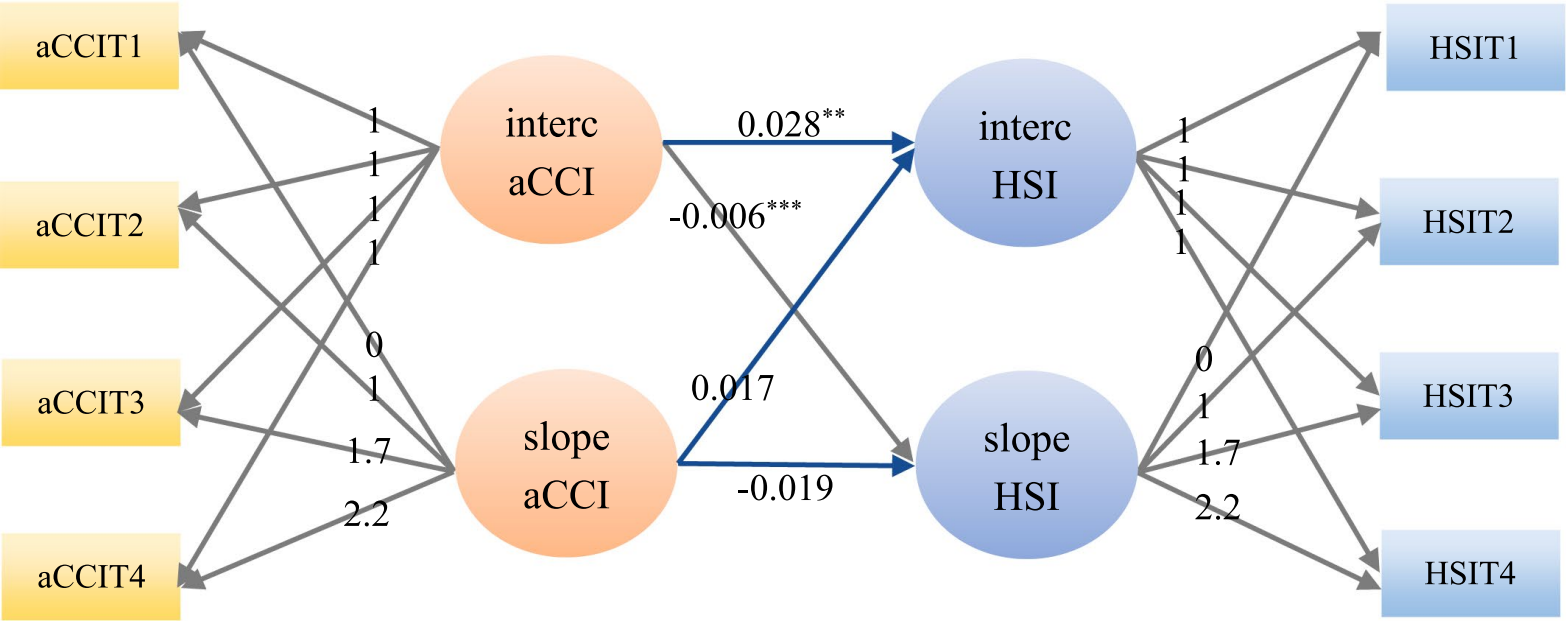
A parallel latent growth model of chronic comorbidities at the health shock level. aCCI, age-adjusted Charlson comorbidity index; HSI, health shock intensity; aCCIT1, age-adjusted Charlson comorbidity index in 2009; aCCIT2, age-adjusted Charlson comorbidity index in 2015; aCCIT3, age-adjusted Charlson comorbidity index in 2019; aCCIT4, age-adjusted Charlson comorbidity index in 2022; HSIT1, health shock intensity in 2009; HSIT2, health shock intensity in 2015; HSIT3, health shock intensity in 2019; HSIT4, health shock intensity in 2022; ^***^p < 0.01, ^**^p < 0.05.

**Figure 7:**
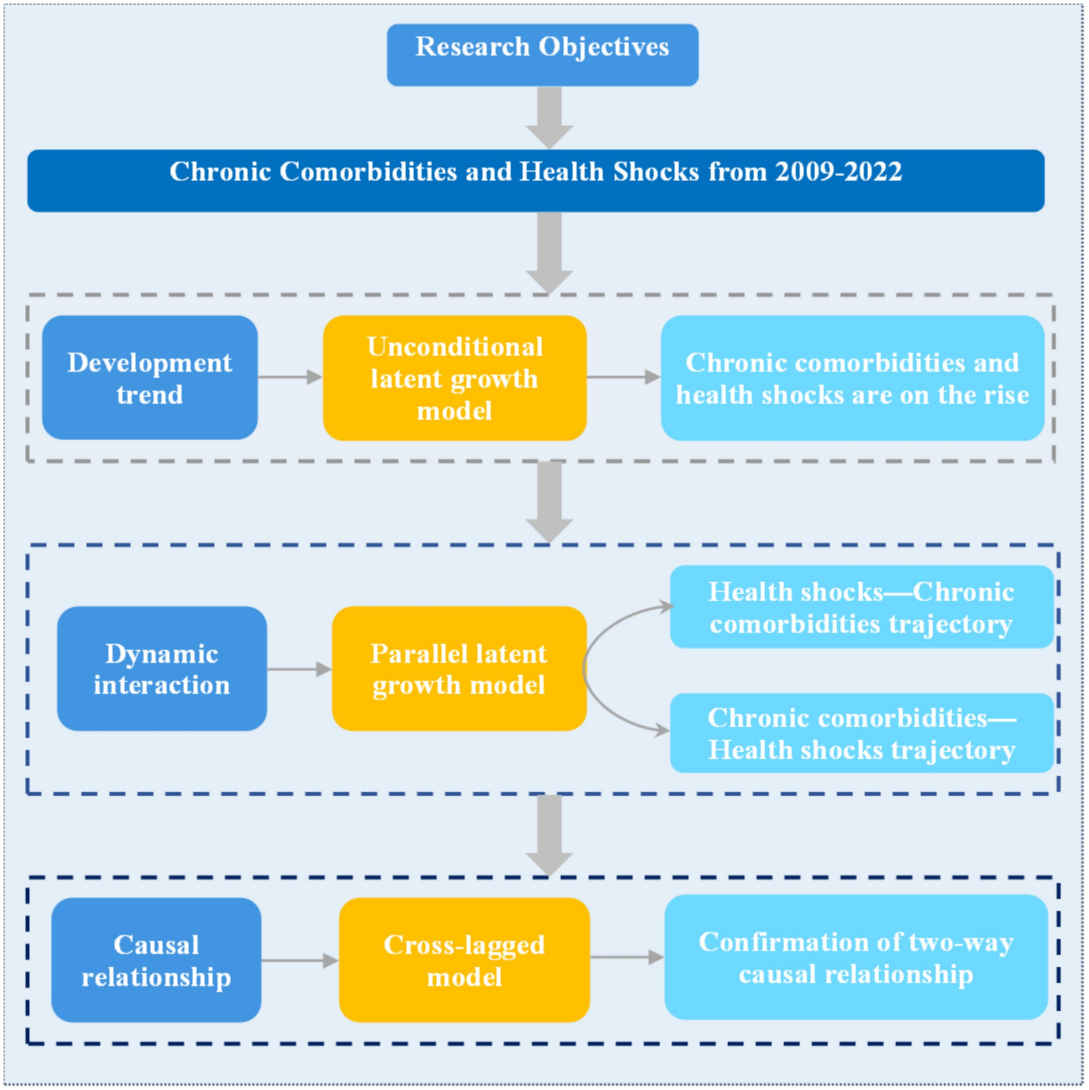
Model path orientation map.

To explore the relationships among the initial level of chronic comorbidities, the developmental trajectory, the initial level of health shock and the developmental trajectory, a PLGM was constructed. First, a PLGM of health shock in the chronic comorbidity direction was constructed, and the model fit well (*χ*^2^ = 104.840, RMSEA = 0.055, SRMR = 0.047, CFI = 0.955, TLI = 0.948). The regression coefficients from the intercept factor of health shock to the intercept factor and slope factor of chronic comorbidity were 6.303 (*p* < 0.05) and 0.217 (*p* < 0.05), respectively, indicating that the initial level of health shock had a significant positive predictive effect on both the initial level and the rate of change of chronic comorbidities. The regression coefficients of the slope factor of health shock to the intercept factor and slope factor of chronic comorbidity were 0.554 (*p* < 0.05) and −0.720 (*p* < 0.05), respectively. It indicates that the rate of change of health shock has a significant positive predictive effect on the initial level of chronic comorbidity, while it has a significant negative predictive effect on the rate of change of chronic comorbidity. As shown in Figure 5. A PLGM of chronic comorbidities at the health shock level was subsequently constructed, and the model fit well (*χ*^2^ = 103.567, RMSEA = 0.058, SRMR = 0.046, CFI = 0.955, TLI = 0.943). The regression coefficients from the intercept factor of chronic comorbidity to the intercept factor and slope factor of health shocks were 0.028 (*p* < 0.05) and −0.006 (*p* < 0.05), respectively, indicating that the initial level of chronic comorbidity had a significant positive predictive effect on the initial level of health shocks and a significant negative predictive effect on the change rate of health shocks. The regression coefficients from the slope factor of chronic comorbidity to the intercept factor and slope factor of health shocks were 0.017 (*p* > 0.05) and −0.019 (*p* > 0.05), respectively, indicating that the rate of change of chronic comorbidity had no significant predictive effect on the initial level and rate of change of health shocks, as shown in Figure 6. Furthermore, this section serves as a transitional link, building upon previous content in a progressive manner. Therefore, a path diagram was constructed to bridge the unconditional latent growth model discussed earlier and the cross-lagged model that follows, as shown in Figure 7.

### 3.5 Cross-lagged model analysis

**Figure 8:**
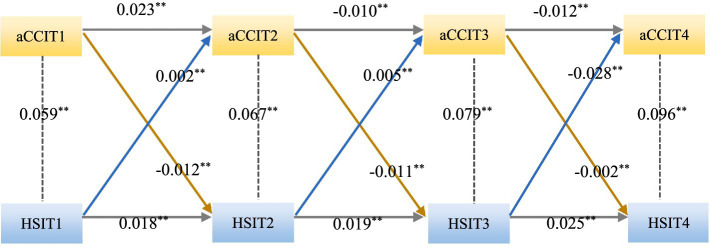
Cross-lagged model for chronic comorbidities and health shock. aCCI, age-adjusted Charlson comorbidity index; HSI, health shock intensity; aCCIT1, age-adjusted Charlson comorbidity index in 2009; aCCIT2, age-adjusted Charlson comorbidity index in 2015; aCCIT3, age-adjusted Charlson comorbidity index in 2019; aCCIT4, age-adjusted Charlson comorbidity index in 2022; HSIT1, health shock intensity in 2009; HSIT2, health shock intensity in 2015; HSIT3, health shock intensity in 2019; HSIT4, health shock intensity in 2022; ^***^p < 0.01; ^**^p < 0.05.

Table 4 and Figure 8 show a CLM of chronic comorbidity and health shock established at three time points with good fitting results (CFI and TLI > 0.9, RMSEA and SRMR < 0.08). The CLM analysis revealed that chronic comorbidities and health shocks were significantly correlated across all four measurements (*p* < 0.05), and health shocks at the second measurement (*β* = −0.012, *p* < 0.05) and chronic comorbidities at the first measurement (*β* = 0.023, *p* < 0.01) were significantly predictive of chronic comorbidities at the first measurement. Health shock at the first measurement significantly predicted health shock at the second measurement (*β* = 0.018, *p* < 0.05) and chronic comorbidity (*β* = 0.002, *p* < 0.05). Health shock at the third measurement (*β* = −0.011, *p* < 0.05) and chronic comorbidity at the second measurement (*β* = −0.010, *p* < 0.05) were significantly predictive of chronic comorbidity at the second measurement. The health shock at the second measurement had a significant predictive effect on the health shock at the third measurement (*β* = 0.019, *p* < 0.05) and chronic comorbidity (*β* = 0.005, *p* < 0.05). Health shock at the fourth measurement (*β* = −0.002, *p* < 0.05) and chronic comorbidity at the third measurement (*β* = −0.012, *p* < 0.05) were significantly predictive of chronic comorbidity at the third measurement. The health shock at the third measurement had a significant predictive effect on the health shock at the fourth measurement (*β* = 0.025, *p* < 0.05) and chronic comorbidity (*β* = −0.028, *p* < 0.05).

**Table 4:** Cross-lagged model for chronic comorbidities and health shocks.

Model fit indices					
*χ* ^2^	*df*	CFI	TLI	RMSEA	SRMR
9.069	12	1.000	1.132	0.000	0.009

## 4 Discussion

### 4.1 During the 14-year follow-up period, chronic comorbidities and health shock tended to increase overall

#### 4.1.1 During the 14 years from 2009 to 2022, chronic comorbidity showed a significant upward trend

With the acceleration of population aging and changes in the mode of chronic disease in China, the coexistence of multiple chronic diseases has become common among middle-aged and older adults people, and the prevalence of chronic comorbidities has increased, resulting in an increasing trend in chronic comorbidities. This research result is similar to that of previous relevant studies (20, 21).

#### 4.1.2 During the 14 years from 2009 to 2022, health shock showed a significant upward trend

As shown in Figure 4, the health shocks showed an overall upward trend, but its model fitting results were poor. The health shocks in 2015 showed a relatively large decline, which is speculated to be related to the implementation of the new medical reform in 2009 and the strong support of poverty alleviation policies during the promotion period of health and poverty alleviation in 2015. In 2009, the new medical reform was implemented. By 2015, after several years of vigorous implementation of the new medical reform, the medical insurance policy had become more complete and the medical level had significantly improved, thereby reducing the health shock risk caused by high medical expenses for families with chronic diseases. Therefore, the level of health shock will have a significant decline in 2015. The research results are similar to those of Xiao et al. (22–24).

In 2019, there was a significant increase, which may be attributed to the outbreak of the novel coronavirus epidemic at the end of 2019. Middle-aged and older adults people in Ningxia suffered a strong health shock in a short period of time, and there was a lack of effective countermeasures to improve the health status of rural middle-aged and older adults people in Ningxia. In addition, middle-aged and older adults people were older, their physical functions declined, and their ability to cope with health shocks decreased. Therefore, in 2019, there was a relatively significant increase.

The year 2022 will be the transition period between rural revitalization and health poverty alleviation. As the country further promotes the implementation of health poverty alleviation policies and the development of rural revitalization strategies, the accessibility of medical services for rural residents will further improve, and medical facilities will improve. For example, the preferential policy of serious illness insurance, the policy of contract services for family doctors and the standardized construction policy of village clinics can improve the health status of rural residents, especially sensitive groups such as middle-aged and older adults individuals in rural areas. Therefore, during the transition period of rural revitalization and health poverty alleviation in 2022, the health impact on rural residents in Ningxia decreased compared with that in 2019. This research can be found in the study of Wang Yanfang et al. (25, 26). Therefore, from the early stage of the new medical reform in 2009 to the transition period of rural revitalization and health poverty alleviation in 2022, which lasted 14 years and four different stages, the health status of middle-aged and older adults people in Ningxia has undergone obvious changes, the degree of change in health impact has fluctuated greatly, and the model fitting result is poor.

### 4.2 There is a dynamic interaction between health shock and chronic comorbidity

#### 4.2.1 The initial level of health shock has a significant positive predictive effect on the initial level and change rate of chronic comorbidity

The greater the initial level of health shock is, the greater the initial level of chronic comorbidities. There is a significant association between chronic comorbidities and the occurrence of health shock, which has been confirmed in several studies (12, 27, 28). The greater the initial level of health shock is, the greater the severity of the health problem or health condition experienced by the individual at a certain point in time. Severe health shocks can lead to reduced body function and a compromised immune system, increasing individuals’ vulnerability to other diseases, and can affect individuals’ medical compliance (such as taking medications on time, having regular check-ups, etc.), thereby increasing the risk of chronic comorbidities. Therefore, the greater the initial level of health shock is, the greater the initial level of chronic comorbidities.

The higher the initial level of health shock is, the faster the rate of comorbidity change of chronic diseases will be. The reason for this may be related to the fact that health shock (such as acute hospitalization) may cause a long-term increase in cortisol by continuously activating the hypothalamic–pituitary–adrenal axis (HPA axis), accelerate chronic inflammation, and thereby promote the decline of multiple organ functions (29). It may also be related to the accumulation of therapeutic side effects. Intensive treatment after health shock may directly induce new chronic diseases (such as diabetes and osteoporosis), manifested as an increasing slope of comorbidity.

#### 4.2.2 The rate of change in health shocks has a significant positive predictive effect on the initial level of chronic comorbidity, while demonstrating a negative predictive effect on its rate of change

The higher the rate of change of health shock, the higher the initial level of chronic comorbidity. Individuals with a high rate of change in health shock tend to utilize medical resources more frequently. This may enable it to be diagnosed with multiple chronic diseases earlier, seemingly presenting a high initial level of chronic comorbidity. Meanwhile, frequent medical visits also reflect unstable health conditions and an increased possibility of coexistence of multiple diseases.

In theory, the change rate of health shock has a significant positive predictive effect on the change rate of chronic comorbidities, but the results of this study show that the change rate of health shock has a significant negative predictive effect on the change rate of chronic comorbidities; that is, the faster the change rate of health shock is, the slower the change rate of chronic comorbidities. The reason may be that when an acute health shock occurs, some interventions are taken accordingly, such as emergency medical interventions, surgery or first aid measures, which can quickly control the condition and thus prevent or slow the development of chronic comorbidities in the short term. After experiencing acute health shock, people tend to pay more attention to their health status and change their negative behaviors, thus slowing the occurrence of health shock. Alternatively, an individual experiencing an acute health shock may seek professional medical help, and in the process, the doctor may identify and treat a chronic disease or other health concern that would otherwise go unnoticed, thereby preventing further deterioration of the health condition; alternatively, after experiencing an acute health shock, individuals will experience a period of psychological stress response and then adapt to changes, such as learning coping skills and enhancing mental toughness, and these changes will help reduce the impact of future health shocks. This conclusion is similar to the results of relevant studies (30, 31).

#### 4.2.3 The initial level of chronic comorbidity has a positive predictive effect on the initial level of health shock and a negative predictive effect on its change rate

The higher the initial level of chronic comorbidity is, the greater the initial level of health shock. The initial level of chronic comorbidity refers to the number and severity of chronic diseases that an individual has prior to a health shock (32). The greater the initial level of chronic comorbidities is, the greater the initial level of health shock, possibly because the presence of chronic comorbidities weakens the immune system, increasing the vulnerability of individuals to infections or other health problems. This may also be related to the constant psychological stress and anxiety that people with chronic diseases often face, which can reduce their quality of life and affect their ability to cope with health shocks. Alternatively, because the treatment costs of patients with chronic diseases are usually relatively high, once they encounter a health shock, they face greater economic pressure, which affects not only the choice of treatment but also the quality of life of patients. The above reasons are related to the higher initial level of chronic comorbidities, the greater the initial level of health shock.

The higher the initial level of chronic comorbidity is, the slower the rate of change in health shock. Individuals with more or more severe chronic comorbidities are likely to deteriorate more quickly and recover more slowly after a health shock than those with no or fewer chronic comorbidities. The possible reason is that individuals with chronic comorbidities have felt tired and mental health pressure due to long-term disease management, and the appearance of health shock may further increase their mental burden, affect their medical compliance behavior and life attitudes, and thus adversely affect their health recovery. At the same time, patients with chronic comorbidities usually face higher medical costs and economic pressure, and the arrival of health shocks undoubtedly exacerbates this burden, which may limit their ability to obtain high-quality medical services and affect treatment effectiveness and health recovery, so the speed of change is slower.

### 4.3 There is a bidirectional causal relationship between chronic comorbidities and health shocks

There is a bidirectional causal relationship between chronic comorbidities and health shocks, where the two entities influence each other and exhibit dynamic correlation. The presence of chronic comorbidities increases an individual’s likelihood of experiencing new health shocks, reduces an individual’s ability to resist disease, and may affect treatment decisions and effectiveness (33). Health shocks can trigger or exacerbate existing chronic diseases (34), leading to long-term health consequences for individuals. Even if the health shock itself is contained, it can leave behind long-term sequelae that can become new sources of chronic disease and promote chronic comorbidities. Health shocks can also cause severe psychological stress, economic burdens and changes in social support networks, indirectly contributing to the development of chronic diseases and thus increasing the burden of chronic comorbidities.

This bidirectional causality creates a vicious circle in which the presence of chronic comorbidities makes an individual more vulnerable to health shocks, and each health shock may add new chronic diseases or exacerbate existing chronic comorbidities, further reducing an individual’s health status and increasing the risk of more health shocks in the future. Therefore, interrupting this cycle, preventing the occurrence and development of chronic comorbidities through integrated management strategies, and effectively responding to health shocks are important for improving public health and reducing pressure on healthcare systems.

## 5 Conclusion

Based on the longitudinal follow-up data from 2009 to 2022, this study analyzed the dynamic correlation between chronic comorbidities and health shocks using the latent growth model and cross-lagged model, and drew the following conclusions: First, the occurrence of chronic comorbidities and health shocks shows a significant upward trend; Second, there is a dynamic interaction between the chronic comorbidities and health shocks. The initial level of health shocks positively predicts the initial level and change rate of chronic comorbidities, and its change rate positively predicts the initial level of chronic comorbidities, but negatively predicts the change rate of chronic comorbidities. The initial level of chronic comorbidities positively predicts the initial level of health shocks, but negatively predicts its rate of change. Third, the cross-lagged model supports the bidirectional predictive relationship between chronic comorbidities and health shocks, indicating that the developmental trajectories of the two influence each other and form a dynamic vicious cycle.

### 5.1 Limitations

This study used the four phases of health follow-up data from 2009, 2015, 2019 and 2022 to explore the developmental trajectory and interaction between chronic comorbidities and health shocks in rural residents in the southern mountainous area of Ningxia, which can provide certain data support for subsequent relevant studies; however, several shortcomings remain. The follow-up data of the four periods of 14 years (2009, 2015, 2019 and 2022) were selected for this survey. A long follow-up time will lead to loss of follow-up and recall bias in the survey population. Although we controlled for the consistency of the survey, such bias was unavoidable, and the interval of follow-up years was uneven. This may affect the results.

## Data Availability

The original contributions presented in the study are included in the article/supplementary material, further inquiries can be directed to the corresponding authors.
